# The genus *Tinginotum* Kirkaldy, 1902 (Hemiptera, Miridae, Mirinae) with description of a new species from Vietnam and additional notes on Southeast Asian materials

**DOI:** 10.3897/zookeys.1269.180930

**Published:** 2026-02-13

**Authors:** Junggon Kim, Frédéric Chérot, Quoc Toan Phan, Minh Ty Nguyen, Ba Vu Lam Nguyen, Sunghoon Jung

**Affiliations:** 1 The Center for Entomology & Parasitology Research, College of Medicine and Pharmacy, Duy Tan University, Da Nang 550000, Vietnam Institute of Applied Technology, Thu Dau Mot University Thu Dau Mot Vietnam; 2 Département de l’Etude du Milieu Naturel et Agricole, Service Public de Wallonie, Gembloux, BE-5030, Belgium Department of Smart Agriculture Systems, College of Agriculture and Life Sciences, Chungnam National University Daejeon Republic of Korea; 3 Institute of Applied Technology, Thu Dau Mot University, No. 06 Tran Van On, Phu Hoa Ward, Thu Dau Mot, Binh Duong, Vietnam Laboratory of Systematic Entomology, Department of Applied Biology, College of Agriculture and Life Sciences, Chungnam National University Daejeon Republic of Korea; 4 Science and International Cooperation Division, Bach Ma National Park, Phu Loc, Hue, Vietnam The Center for Entomology & Parasitology Research, College of Medicine and Pharmacy, Duy Tan University Da Nang Vietnam; 5 Laboratory of Systematic Entomology, Department of Applied Biology, College of Agriculture and Life Sciences, Chungnam National University, Daejeon, Republic of Korea Département de l’Etude du Milieu Naturel et Agricole, Service Public de Wallonie Gembloux Belgium; 6 Department of Smart Agriculture Systems, College of Agriculture and Life Sciences, Chungnam National University, Daejeon, Republic of Korea Science and International Cooperation Division, Bach Ma National Park Hue Vietnam

**Keywords:** New species, plant bugs, Southeast Asia, taxonomy, true bugs

## Abstract

The plant bug genus *Tinginotum* Kirkaldy is reviewed from Vietnam. Four species are recognized, including a new species, *Tinginotum linhi* Kim, Chérot & Jung, **sp. nov**., and new distributional records of *T. bilineatum* and *T. perlatum* from Vietnam. Detailed diagnoses, descriptions, and a key to the Vietnamese species are provided. Male and female genitalia of *T. bilineatum* and female genitalia of *T. knowlesi* are described and illustrated for the first time. In addition to reviewing the Vietnamese fauna, distributional records from neighboring countries were also examined, leading to updated occurrence data for the genus. A particular focus is given to the potential broad-range distribution of *T. perlatum*, considering whether this pattern reflects a truly widespread species or unresolved conspecificity with taxa described from tropical regions. Furthermore, for several Southeast Asian species that were described long ago, the early 19^th^ century, but remain poorly known, we provide photographs of the type specimens and summarize the essential key characters based on the images examined and the original descriptions. These notes are intended to encourage future comprehensive taxonomic research on the genus and its related taxa.

## Introduction

The genus *Tinginotum* was described by Kirkaldy (1902) to accommodate the type species by monotypy *T. javanum* from Java, Indonesia. African species were later studied in detail by Odhiambo (1960), the species from Papua New Guinea being treated by Carvalho (1987) and listed by Chérot et al. (2017), the species from Japan by Yasunaga (1999), the species from China by Zheng et al. (2004) and the species from Australia and New Zealand by Eyles (2000). Regardless of these different works, the genus remains in great need of revision on a worldwide scale.

Continuing ongoing effort to document the specific richness of plant bugs of subfamily Mirinae (Insecta: Heteroptera: Miridae) from Vietnam (Kim et al. 2025a, 2025b), the present paper provides the description of a new species, *Tinginotum linhi* sp. nov. from Phu Loc, Hue City, and mentions two additional species from the country for the first time: *T. bilineatum* Zheng & Lu, 2002 and *T. perlatum* Linnavuori, 1961. The latter species is also reported for the first time from Indonesia (Sumatra) and the Philippines (Luzon, Mindanao), whereas *T. knolwesi* (Kirkaldy, 1908) is newly recorded from Cambodia.

Each Vietnamese species is diagnosed and described or redescribed with documentation of external morphology and genitalia of both sexes. To facilitate future studies of the genus, illustrations of type specimens of some other Oriental species and a key to Vietnamese species are also provided.

## Materials and methods

The photographs of the specimens were taken using an Optinity digital camera mounted on a ZEISS Stemi 508 microscope, with image stacks processed using Helicon Focus 8 software. Final figures were prepared and edited in Adobe Photoshop 2020 and Affinity Photo 2. Measurements, reported in millimeters (mm), were obtained with OptiView software on the same camera system. Male and female genitalia were examined after detaching and macerating the abdomen in a 10% KOH solution at 70 °C for five minutes, until the internal structures were visible. The type specimens of the new species *T. linhi* sp. nov. are deposited in the Zoological Collection of Duy Tan University, Da Nang, Vietnam (**ZCDTU**). Depositories of other specimens are abbreviated as:

**CNU**: Laboratory of Systematic Entomology, Chungnam National University, Daejeon, Korea;

**FMNH**: Finnish Museum of Natural History, Helsinki, Finland;

**ISNB**: Institut royal des Sciences naturelles de Belgique, Département d’Entomologie, Brussels, Belgium;

**NHMW**: Naturhistorisches Museum Wien, Austria.

The terminology for external and genital morphological structures primarily follows Chérot et al. (2025). The asterisk (*) following a locality indicates that the species is newly recorded from that region.

## Results

### Taxonomy

#### Genus Tinginotum

Taxon classificationAnimaliaHemipteraMiridae

Kirkaldy, 1902

8CA2101A-FB3E-5C74-B010-9AD8CE49F8DC

Tinginotum Kirkaldy 1902: 263. Type species: Tinginotum javanum Kirkaldy, 1902. Odhiambo 1960: 465; Carvalho 1987: 169; Yasunaga 1999: 39; Eyles 2000: 111; Zheng et al. 2004: 720; Chérot et al. 2025: 243.
Hermotinus Distant, 1904: 462 (syn. by Carvalho 1952: 93). Type species: Hermotinus signatus Distant, 1904.
Nesodaphne Kirkaldy, 1908: 380 (syn. by Carvalho 1987: 166). Type species: Nesodaphne knowlesi Kirkaldy, 1908.
Eutinginotum Cheesman, 1926: 266 (syn. by Cheesman 1927: 157). Type species: Eutinginotum raiateae Cheesman, 1926 (= Nesodaphne knowlesi Kirkaldy, 1908).

##### Diagnosis.

(summarized and adapted from Chérot et al. 2025). Elongate-oval, relatively small to medium-sized (total length 3.2–6.8) plant bugs, dorsally yellowish, orange, brown and reddish-brown or greenish with silvery pruinose areas on hemelytra, sometimes fuzzy or almost totally absent; dorsum with vestiture composed of several types of setae, predominant dorsal setae rounded in cross section, elongate, relatively stiff, suberect to erect, sometimes in tufts, golden, silvery or dark brown to black, in addition some species with tufts of recumbent, white setae arising from pale spots; head short, declivous; vertex carinate, finely sulcate or without sulcus; maxillary plate not tuberculate; first antennal segment relatively thick and generally elongate, widened sub-basally and apically; second antennal segment elongate, almost cylindrical; labium reaching meso- or metacoxae; pronotal collar longer than first antennal segment maximal width; pronotum devoid of lateral carina, almost trapezoidal, humeral angles rounded, lateral margins almost straight to slightly concave, posterior margin almost straight to convex; callosities reduced, medially separated and reaching pronotal lateral margins; punctation of pronotal disk clear and generally deep; disk medially devoid of hump; mesoscutum covered or almost covered; scutellum slightly swollen, with smaller and shallower punctation than that in pronotum; hemelytral punctation reduced, a line of shallow but relatively wide punctures present along claval suture and R+M vein; membrane and cuneus slightly deflexed; metatibial spines present; endosoma with three main lobes, sometimes with two elongate, apically pointed sclerites, a ctenoidal process and a sclerite associated with secondary gonopore; parieto-vaginal rings relatively small, rounded, distinctly separated, devoid of additional sclerite; anterior, latero-inner and frequently latero-outer margins convex, posterior margin concave to almost straight, without sclerite between rings; posterior wall elongate, with a wide dorsal structure; sigmoid process complex but reduced or apparently absent; inter-ramal sclerites wide, totally separated medially; inter-ramal lobes wide, covering in dorsal view almost 2/3 of inter-ramal sclerites or more; lateral lobes missing.

##### Remarks.

Several genera that are morphologically very similar to *Tinginotum*, including *Argenis* Distant, 1904, *Tinginotopsis* Poppius, 1915, and *Diognetus* Distant, 1904, have been regarded as closely related by previous authors (Yasunaga 2023; Yasunaga et al. 2023; Chérot et al. 2025). Recent taxonomic changes, such as the transfer of a species formerly placed in *Tinginotum* to *Argenis* (Yasunaga 2023), indicate that the delimitation of generic boundaries within this group is still under discussion. As currently understood, *Argenis* can be distinguished from *Tinginotum* mainly by the tuberculate maxillary plate (see Chérot et al. (2025) for details for *Argenis*). *Tinginotopsis* is highly similar to *Tinginotum* in most morphological characters and differs mainly in the presence of a pronotal protuberance, which has led to differing interpretations of their generic status (Eyles 2000; Yasunaga 2023; Yasunaga et al. 2023; Chérot et al. 2025).

*Tinginotum* is geographically widespread and morphologically diverse, yet it can be recognized based on the above diagnosis. This genus was recovered as monophyletic in a phylogenetic study (Kim and Jung 2019), and subsequent molecular analyses further suggested a close relationship between *Tinginotum* and *Tinginotopsis* (Namyatova et al. 2021). However, as both studies were conducted with taxon sampling appropriate to their respective study scopes, the monophyly of *Tinginotum* and the boundaries between these two genera as well as relationships with other similar groups remain unclear. Therefore, further analyses incorporating broader taxon sampling would be helpful.

###### Key to the *Tinginotum* species in Vietnam

**Table:** 

1	Pronotum wide, medial length distinctly less than head width (Figs 1A, 1B, 1D, 6A, 6B, 6D–H)	**2**
–	Pronotum elongate, medial length distinctly more than or subequal to head width (Figs 3A, 3C, 4A)	**3**
2	Second antennal segment distinctly bicolored (Fig. 1A–D); clypeus brown, concolorous to frons (Fig. 1H–K); hind tibiae with four distinct dark bands (Fig. 1A–G); left paramere faintly curved; shaft T-shaped (Fig. 2A, B)	***T. linhi* sp. nov**.
–	Second antennal segment gradually darkened or not clearly divided, apex somewhat darker (Fig. 6A, B, D–H); clypeus entirely dark brown (Fig. 6C, I); hind tibiae with dark tiny spots (Fig. 6D–H); left paramere distinctly curved; shaft hook-shaped (Fig. 7A, G)	***T. perlatum***
3	Pronotum with two longitudinal pale stripes medially, medial length distinctly more than head width (Fig. 3A, C); antennae partially brown and dark brown (Fig. 3A–E); scutellum with large greenish marking medially (Fig. 3A, C); femur with dark bands apically (Fig. 3A–E)	***T. bilineatum***
–	Pronotum with darker markings, medial length subequal to head width (Fig. 5A); antennae mostly dark brown with pale bands, base, and apices (Fig. 5A, B); scutellum brown with pale longitudinal stripe (Fig. 5A); femur with dark stripe apically (Fig. 5A)	***T. knowlesi***

#### Tinginotum linhi

Taxon classificationAnimaliaHemipteraMiridae

Kim, Chérot & Jung sp. nov.

59AAE3AF-FAC8-52FE-80C9-7923BAE75FFB

https://zoobank.org/F2C604C4-2FC6-40C1-A16E-83CC49186904

Figs 1, 2

##### Type material.

***Holotype***: • 1♂, Vietnam, Hue City, Phu Loc, Bach Ma National Park (16.1942°N, 107.8631°E), 1356 m altitude, 16.xi.2024, on *Pinus kesiya* Royle ex Gordon, 1858, Kim J. leg. [ZCDTU]; ***paratypes***: • 1♂1♀, same location as holotype, 1250 m altitude, 6.ix.2025, by light trap, Kim J. leg. [ZCDTU].

##### Diagnosis.

Recognized by dorsum brown to dark brown; clypeus brown, concolorous with frons; antennae shorter than body; first antennal segment short, cylindrical, medially weakly narrowed, slightly shorter than 1/2 head width (including eyes); second antennal segment partially brown to dark brown, distinctly dark brown at extreme base and apical 1/3; third segment mostly dark brown except for pale extreme apex; labium slightly exceeding mid coxae; first labial segment mostly pale brown with darker spot; pronotum wide, mostly dark brown, posterior margin pale brown; scutellum mostly dark brown, with pale apex; hemelytra brown with large dark brown markings, appearing overall dark brown; embolium narrow; cuneus semi-hyaline, inner and anterior margins tinged with red; hind femur partially pale to dark brown, basal 1/3 pale, apical 2/3 with dark markings; hind tibia brown with four distinct bands subequal in length; left paramere curved or arched with a basal lobe, shaft short, T-shaped, apex of shaft straight, lower shaft part bifurcate (Fig. 2A, B); endosoma membranous with three sclerites; primary sclerite curved, longer than others, its apex broadened; median sclerite curved, tapered to apex; third lobal sclerite broad with narrow rod-shaped appendages (Fig. 2E–G).

**Figure 1.**
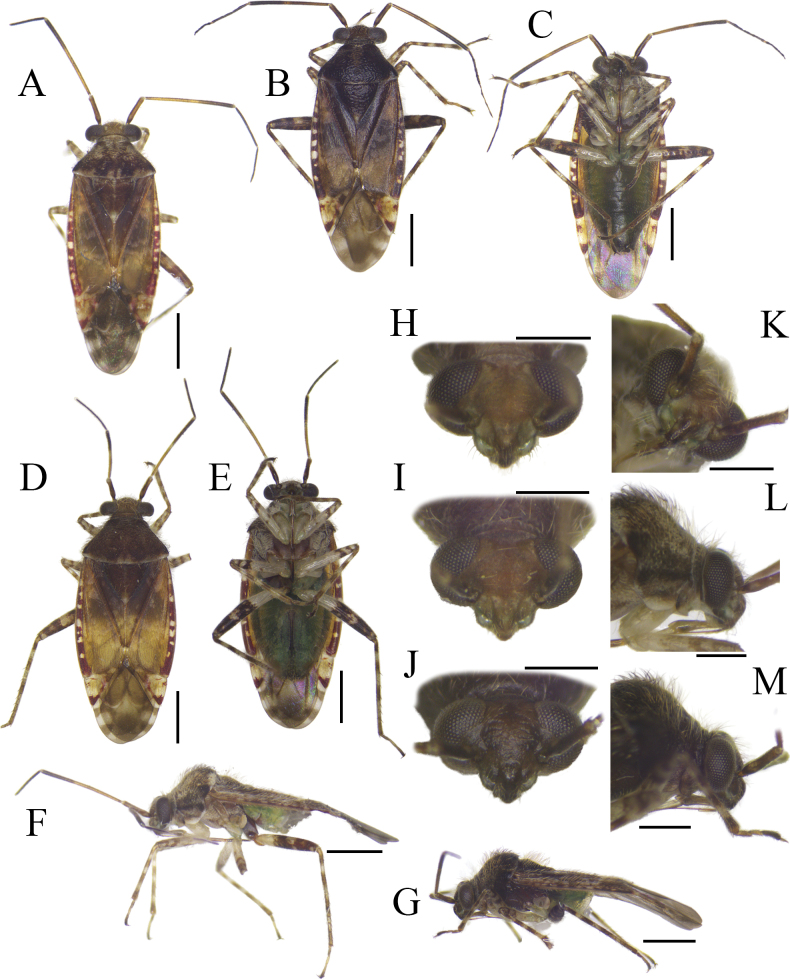
Habitus and diagnostic characters of *Tinginotum linhi* sp. nov. **A**. Holotype, male; **B, C**. Paratype, male; **D, E**. Paratype, female; **F, G**. Holotype and paratype males in lateral view; **H–M**. Head in frontal, frontoventral and lateral views. Scale bars: 1 mm (**A–G**); 0.5 mm (**H–M**).

**Figure 2.**
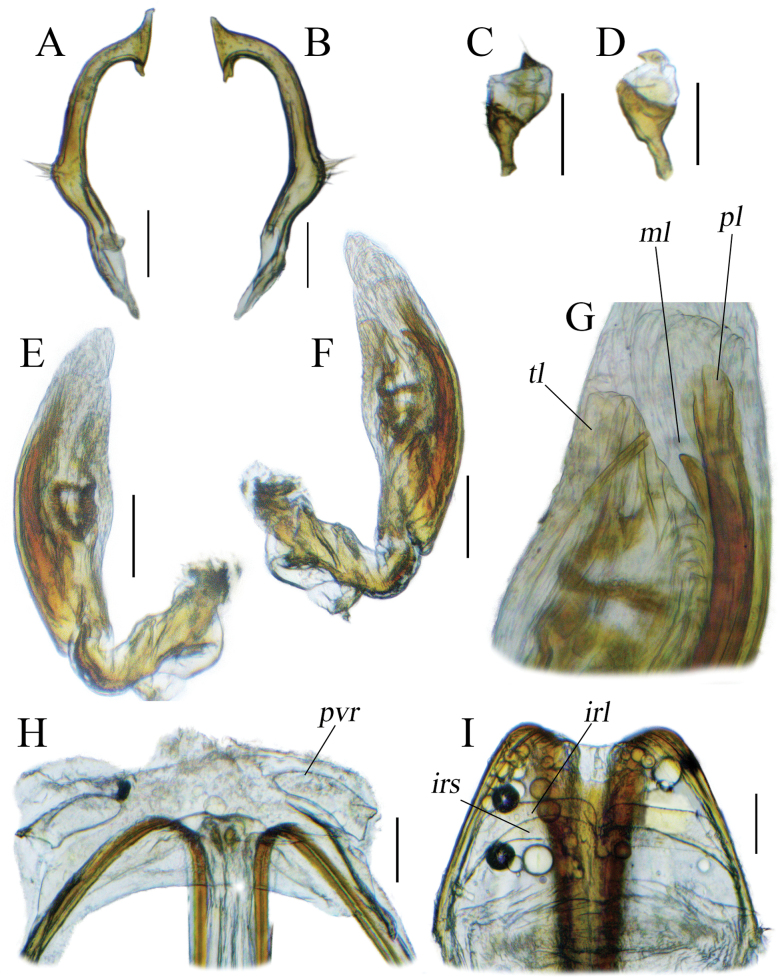
Male and female genitalia of *Tinginotum linhi* sp. nov. **A, B**. Left paramere; **C, D**. Right paramere; **E–G**. Endosoma; **H**. Genital chamber; **I**. Posterior wall. Abbreviations: *irl* = interramal lobe; *irs* = interramal sclerite; *ml* = median sclerite; *pl* = primary sclerite; *pvr* = parieto-vaginal rings; *tl* = third sclerite. Scale bars: 0.1 mm.

##### Description.

**Male: *Coloration***: body varying from brown to fuscous, bearing markings darker than body coloration, partially tinged with red. ***Head***: vertex partially pale brown to brown with a pair of pale spots near eyes, mostly dark brown in dark specimens; frons brown, entirely dark brown in dark individuals; clypeus mostly brown, almost concolorous with frons, comparable in tone to the darkest area of mandibular plate, mostly dark brown in dark individual; mandibular plate paler, dark brown in dark specimens; maxillary plate mostly pale brown; antennae partially brown to dark brown; first segment brown, base and inner lateral margin dark brown, base, inner lateral margin and ventral part dark brown in dark specimens; second segment pale brown to dark brown, base and apical 1/3 part dark brown, apex pale brown; third and fourth segments entirely dark brown except for pale apex of third segment; labium partially pale to dark brown; first segment pale brown with darker spot subapically, mostly brown with darker spot subapically in dark specimens; third segment pale brown, basally dark brown, basally dark brown in dark specimens; fourth segment dark brown. ***Thorax***: pronotum brown with dark markings and pale brown posterior margin, mostly dark brown with pale posterior margin in dark specimens; propleuron, mesepisternum, mesepimeron and metepimeron brown, mostly dark brown in dark specimens; scent efferent system pale brown and brown; scutellum mostly dark brown, longitudinal medial pale band and pale apex, mostly dark brown with pale apex in dark specimens; hemelytra brown with large dark markings in medial part and with small pale spots in general; corium generally brown with darker marking medially and posteriorly, and minute pale spots scattered irregularly, with lower density of spots in central area, making the darker spots relatively larger; clavus mostly dark brown with few tiny pale scattered spots; embolium subhyaline with irregularly distributed reddish markings, with dark markings tinged with red in dark specimens; cuneus generally subhyaline with dark markings on inner and posterior areas, tinged with red at inner and outer margins, small area tinged with red medially; legs pale brown with dark markings and bands; fore and mid femora with dark markings medially and two bands apically; hind femora with dark markings at 2/3 apical part, basal 1/3 pale; tibiae pale with four dark bands, two bands at base and apex, two bands at middle, bands nearly equal in length, with first band slightly shorter at base; all tarsi partially brown to dark brown; first and third tarsal segments dark brown. **Abdomen**: greenish brown. ***Surface and vestiture***: body dull, densely covered with three types of setae; two types long and erect, golden or dark in color, and a third type consisting of short, recumbent white tufts originating from pale spots; pronotum and scutellum punctate, scutellum weakly punctate; hemelytra impunctate, except for row of punctures along claval vein, row along R+M, and row along costal vein.

***Structure***: body elongate, length 4.57–4.76. **Head**: mostly hypognathous, 2.5 × as wide as long; vertex 0.88 × as wide as single compound eye width; antennae generally linear, with varying thickness, shorter than body length; first segment cylindrical, twice as thick as second segment, 1.53 × as long as vertex width, 0.28 × as long as second segment; second segment 1.15 × as long as combined third and fourth segments length; third segment 1.29 × as long as fourth segment; proportion of first to fourth antennal segments 0.49: 1.74: 0.85: 0.66; labium slightly exceeding mid coxae. **Thorax**: pronotum trapezoid, elevated laterally, dorsal longitudinal length 0.55 × as long as basal maximal width, 0.77 × pronotal height in lateral view, lateral margin weakly constricted near calli, posterior margin broadly convex, weakly sinuate medially; calli weakly swollen; pronotal collar broad, broader than first antennal segment maximal diameter; scutellum swollen, width subequal to length, subequal to claval commissure; exposed part of mesoscutum narrow; lateral margins of hemelytra rounded; cuneal outer margin 0.31 × as long as embolial margin; fore leg with second tarsomere short; mid and hind legs with first tarsomere short, third tarsomere longest. **Abdomen**: tapering to apex, almost reaching to apex of cuneus. ***Genitalia***: left paramere weakly curved, shaft short, T-shaped, apex of shaft straight, downward part bifurcate, body long, sensory lobe weakly developed, covered with tufts of setae (Fig. 2A, B); right paramere short, stout, apex of shaft beak-like (Fig. 2C, D); endosoma membranous with three sclerites; primary sclerite (*pl*) curved, longest, its apex broadened; median sclerite (*ml*) curved, tapered to apex, shortest; third lobal sclerite (*tl*) broad, associated to secondary gonopore, with narrow rod-shaped appendages (Fig. 2E–G).

**Female: *Coloration***: as in male except for second antennal segment brown and dark brown with small pale spot in dorsal side. ***Surface and vestiture***: as in male. ***Structure***: as in male, except for larger body, length 4.96 mm, and slightly longer third antennal segment, length 0.96 mm. ***Genitalia***: genital chamber with parieto-vaginal rings (*pvr*) elongate oval, inner margin rounded, outer margin somewhat concave with two tapered apices (Fig. 2H); posterior wall with relatively simple interramal sclerite (*irs*) and interramal lobe (*irl*); *irs* narrow, separated medially, narrowed from subapical regions to apex; *irl* broad and wide, entirely covering *irs* and extending further, separated medially, but closed each other (Fig. 2I).

##### Measurements.

Male (*n* = 2)/Female (*n* = 1). Body length, clypeus–apex of membrane: 4.57–4.76/4.96; head length, excluding collar: 0.41–0.42/0.41; head width, including compound eyes: 1.03–1.08/1.10; vertex width: 0.31–0.33/0.39; first antennal segment length: 0.48–0.50/0.49; second antennal segment length: 1.70–1.78/1.70; third antennal segment length: 0.85 × 0.86/0.96; fourth antennal segment length: 0.65–0.67/missing; total antennal length: 3.66–3.81/3.15 (first–third); mesial pronotal length in dorsal view: 0.85–0.88/0.95; posterior pronotal maximal width (straight): 1.51–1.61/1.75; anterior scutellar width: 0.81–0.86/0.98; mesial scutellar length: 0.82–0.87/0.87; claval commissure length: 0.84–0.85/0.95; maximal width across hemelytron: 0.86–0.96/0.99.

##### Etymology.

The specific epithet is named linhi, honoring Dr. Nguyen Vu Linh, Director of Bach Ma National Park, for his support and dedication to wildlife conservation in Vietnam. The name is a noun in the genitive case.

##### Plant association.

*Pinus kesiya* Royle ex Gordon, 1858 (Pinaceae).

##### Distribution.

Vietnam (central).

##### Remarks.

*Tinginotum linhi* sp. nov. can be easily distinguished from several superficially similar species of the genus by the following character states:

- Compound eyes relatively large vs small, their width inferior to vertex width [e.g., *T. galleni* (Poppius, 1912); *T. vescum* Odhiambo, 1960];
- First antennal segment relatively short vs relatively long proportionally to head width [e.g., *T. bilineatum* Zheng & Lu, 2002; *T. knowlesi* (Kirkaldy, 1908); *T. grandis* Carvalho, 1987];
- First antennal segment slightly vs distinctly swollen at the basal and/or apical portions [e.g., *T. javanum* Kirkaldy, 1902; *T. gracilicorne* Poppius, 1915];
- Second antennal segment mostly pale brown and apical 1/3 part dark brown vs mostly dark brown [e.g., *T. signatum* (Distant, 1904); *T. bipuncticolle* Poppius, 1912; *T. villosulum* (Distant, 1913); *T. formosanum* Poppius, 1915; *T. pini* Kulik, 1965];
- Labium reaching slightly beyond mid coxae vs extending apex of hind coxae or reaching abdomen [e.g., *T. zebrinum* Odhiambo, 1960; *T. rostratum* Kerzhner, 1972];
- Shaft of the left paramere being hook-shaped [e.g., *T. floraensis* Carvalho, 1987; *T. rubrovenosus* Carvalho, 1987; *T. befui* Yasunaga, 1999].

Among the remaining species, this new species is similar to *T. minutum* Eyles, 2000 from Australia and New Zealand, but can be distinguished by the following character states:

- Base of first antennal segment dark brown in dorsal view (vs basal half of first segment totally dark brown);
- 1/3 apical part of second antennal segment dark brown (vs apex of second segment dark brown);
- Clypeus brown, concolorous to frons (vs clypeus dark brown, not concolorous to frons);
- All tibiae brown with distinct dark bands (vs all tibiae without distinct dark band);
- Left paramere faintly curved (vs left paramere distinctly curved); sensory lobe of left paramere weakly developed (vs sensory lobe strongly developed) (see Eyles (2000) for original description with illustrations).

*Tinginotum linhi* sp. nov. is also similar to *T. perlatum* Linnavuori, 1961 (Figs 6, 7), but can be distinguished by the following character states:

- Second antennal segment distinctly bicolored without gradation, 1/3 apical part of second antennal segment dark brown (vs second segment gradually darkened or almost unicolorous);
- Clypeus brown, concolorous to frons (vs clypeus entirely dark brown, not concolorous to frons);
- Pronotum mostly dark brown without pale longitudinal line or markings (vs pronotum with pale longitudinal hourglass-shaped marking, sometimes almost erased);
- Hind tibiae brown with four distinct dark bands (vs hind tibiae brown with dark tiny small spots);
- Left paramere faintly curved (vs left paramere distinctly curved); shaft T-shaped (vs shaft hook-shaped); sensory lobe of left paramere weakly developed (vs sensory lobe strongly developed);
- Primary endosomal sclerite slightly longer than median sclerite (vs primary sclerite much longer than median sclerite).

The new species also resembles *T. kirkaldyi* from Java (Fig. 9A–D), but differs by the same three diagnostic character states (a–c) noted above for distinguishing it from *T. perlatum*.

Finally, *Tinginotum linhi* sp. nov. can be distinguished from *T. virescens* Poppius, 1914 from Java and Papua New Guinea (Fig. 9E–G), by:

- Body mostly brownish coloration (vs body greenish brown);
- Second antennal segment distinctly bicolored without gradation, 1/3 apical part of second antennal segment dark brown (vs second almost unicolorous);
- Pronotum mostly dark brown without pale longitudinal line or markings (vs pronotum with pale longitudinal hourglass-shaped marking);
- Embolium with markings (vs embolium unicolorous, without markings);
- Cuneus with dark marking at apex (cuneus without dark marking apically); and
- All tibiae brown with distinct dark bands (vs all tibiae without distinct dark band).

#### Tinginotum bilineatum

Taxon classificationAnimaliaHemipteraMiridae

Zheng & Lu, 2002

92F44083-78F5-5A9D-BA11-44AD2BE20D4C

Figs 3, 4

Tinginotum bilineatum Zheng & Lu, 2002: 503; Zheng et al. 2004: 585.

##### Material examined.

• 6♂♂11♀♀, Vietnam, Hue City, Phu Loc, Bach Ma National Park (16.2281°N, 107.8583°E), 1250 m altitude, 6.ix.2025, by light trap, *Kim J*. leg. [ZCDTU].

##### Diagnosis.

Recognized by dorsum generally pale brown with dark and greenish brown markings; clypeus generally brown with two longitudinal dark stripes along lateral margins; antennae shorter than body; first antennal segment elongate, more than 3 × vertex width, mostly pale brown with lateral dark stripe; second antennal segment partially brown and dark brown, with distinctly dark brown extreme base and apical 1/5; labium reaching mid coxae; pronotum elongate, brown, with two pale longitudinal stripes medially and two short pale stripes near lateral margin, area between medial stripes brown, posterior margin pale brown; pronotal collar entirely pale brown; scutellum brown with large greenish brown spot medially, apices pale brown; hemelytra pale brown with dark markings; clavus mostly pale brown with dark markings; embolium with dark markings; cuneus semi-hyaline with dark markings at anterior and apical parts; fore and mid femora with dark apical bands; hind femur pale, with dark medial markings and dark apical bands; hind tibia brown with four distinct bands, narrow and nearly equal in length; left paramere strongly curved, shaft long, apex of shaft hook-shaped (Fig. 4A–D); endosoma membranous with three long sclerites; primary sclerite and median sclerite long, apically tapered; apex of third lobal sclerite blunt (Fig. 4F, G).

**Figure 3.**
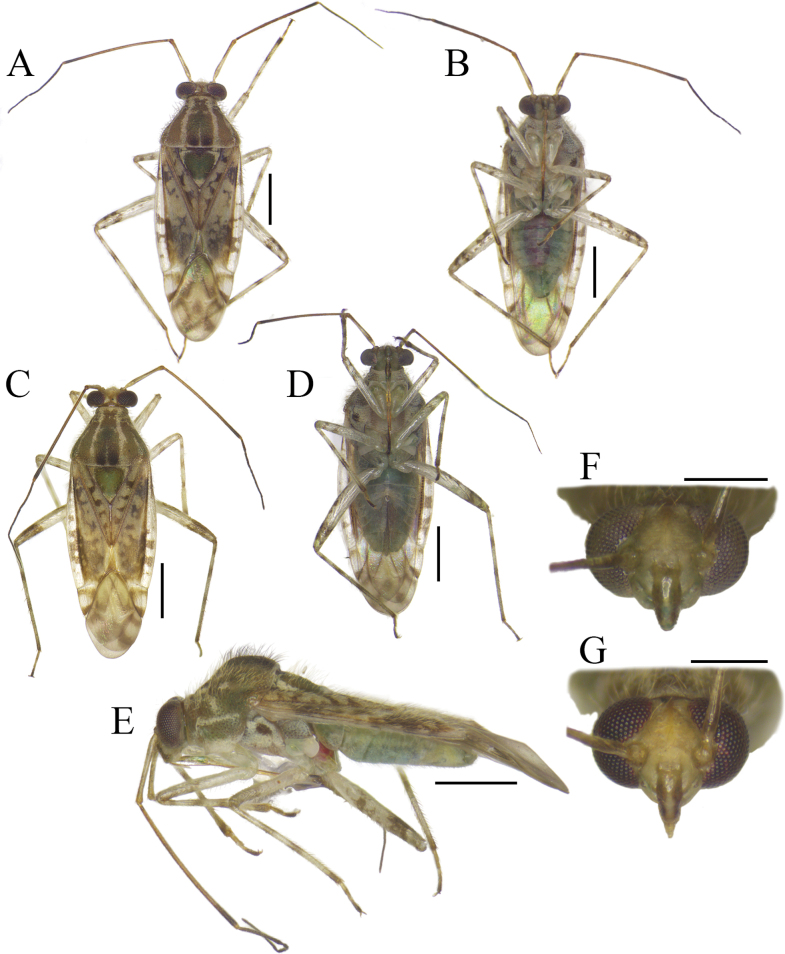
Habitus and diagnostic characters of *Tinginotum bilineatum*. **A–E**. Male and female in dorsal, ventral, and lateral views; **F, G**. Head in frontal view. Scale bars: 1 mm (**A–E**); 0.5 mm (**F, G**).

##### Description.

**Figure 4.**
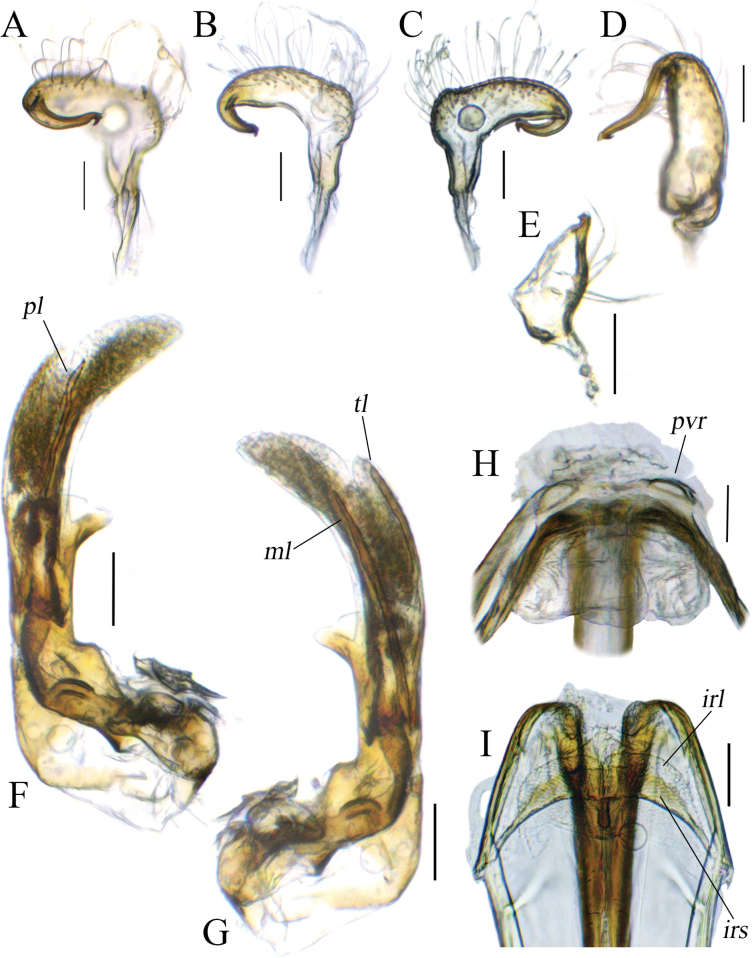
Male and female genitalia of *Tinginotum bilineatum*. **A–D**. Left paramere; **E**. Right paramere; **F, G**. Endosoma; **H**. Genital chamber; **I**. Posterior wall. Abbreviations: *irl* = interramal lobe; *irs* = interramal sclerite; *ml* = median sclerite; *pl* = primary sclerite; *pvr* = parieto-vaginal rings; *tl* = third sclerite. Scale bars: 0.1 mm.

See Zheng and Lu (2002) for original description. Since the information on male and female genitalia was not included in the original description, we provide a description of both here. ***Male genitalia***: Left paramere distinctly curved, covered with long setae except for shaft, shaft curved and long, weakly tapered, apex of shaft hook-shaped, sensory lobe weakly developed (Fig. 4A–D); right paramere stout, shaft short, apex of shaft hook-shaped, outer margin covered with long setae (Fig. 4E); endosoma membranous with three long sclerites; primary sclerite (*pl*) slightly curved subapically, apex sharp; median sclerite (*ml*) almost straight, tapered to apex, third sclerite (*tl*) slightly rounded, apex blunt (Fig. 4F, G). ***Female genitalia***: Genital chamber with parieto-vaginal rings (*pvr*) elongate oval, inner margin somewhat blunt, outer margins tapered, anterior margin of ring thick (Fig. 4H); posterior wall with relatively simple interramal sclerite (*irs*) and interramal lobe (*irl*); *irs* not separated and broadened medially, narrowed from subapical regions to apex; *irl* broad, entirely covering *irs* and extending further, fused medially, gradually narrowed toward apex (Fig. 4I).

##### Measurements.

Male (*n* = 4)/Female (*n* = 5) Body length, clypeus–apex of membrane: 5.05–5.21/5.01–5.40; head length, excluding collar: 0.37–0.44/0.37–0.41; head width, including compound eyes: 0.97–1.01/0.96–0.99; vertex width: 0.23–0.24/0.24–0.27; first antennal segment length: 0.81–0.89/0.79–0.86; second antennal segment length: 1.80–2.19/1.75–2.07; third antennal segment length: 1.09–1.21/0.99–1.20; fourth antennal segment length: 0.63–0.74/0.57–0.70; total antennal length: 4.43–5.03/4.19–4.67; mesial pronotal length in dorsal view: 1.07–1.15/1.08–1.16; posterior pronotal maximal width (straight): 1.53–1.60/1.55–1.63; anterior scutellar width: 0.84–0.87/0.85–0.91; mesial scutellar length: 0.76–0.77/0.71–0.77; claval commissure length: 0.85–0.86/0.84–0.89; maximal width across hemelytron: 0.87–0.91/0.89–0.91.

##### Plant association.

Unknown.

##### Distribution.

China, Vietnam (central)*.

##### Remarks.

As remarked above, previous studies on *Tinginotum* have hypothesized a possible relationship with *Tinginotopsis* (Yasunaga 2023; Yasunaga et al. 2023; Chérot et al. 2025). The two genera have been distinguished solely on the basis of a hump-like protuberance on the pronotum, with no other consistent differences known. In the present study, we found that *Tinginotum bilineatum*, although lacking such a protuberance, is morphologically and anatomically very similar to *Tinginotopsis tuberculatus* Eyles, 2000 (see Eyles 2000: figs 25, 26, 29) and also resembles *Tinginotopsis camelus* Poppius, 1915 and *Tinginotopsis moifensis* Carvalho, 1987 in the structure of the left paramere (see Carvalho 1987: figs 1, 3, 7). Because the paramere structure in *Tinginotum* is diverse and largely comparable to that of *Tinginotopsis*, this character has not been considered diagnostic. Nevertheless, the left paramere of *Tinginotopsis* (except for *Tinginotopsis wauensis* Carvalho, 1987) shows a consistent and distinctive configuration (left paramere three-dimensionally configured; basal part rising vertically, then abruptly bent at a right angle, forming an L-shaped outline in lateral view. From the apical end of this bend, the shaft curves smoothly and rounded inward, producing a U-shaped configuration in dorsal view). In our view, the combination of a *Tinginotum*-type pronotum and a *Tinginotopsis*-type left paramere in *Tinginotum bilineatum* provides additional morphological evidence supporting previous hypotheses that the generic limits between *Tinginotum* and *Tinginotopsis* are uncertain and that a comprehensive revision of these genera is required.

#### Tinginotum knowlesi

Taxon classificationAnimaliaHemipteraMiridae

(Kirkaldy, 1908)

168B793C-6319-5DCB-A1AB-C4A937B30BC2

Fig. 5

Nesodaphne knowlesi Kirkaldy, 1908: 381; Carvalho and Wallerstein 1976: 688.
Tinginotum papuanum Poppius, 1915: 56 (syn. by Carvalho 1987: 174).
Tinginotum cretaceum Poppius, 1915: 58 (syn. by Carvalho 1987: 174).
Eutinginotum raiateae Cheesman, 1926: 267 (syn. by Carvalho 1987: 174).
Nesodaphne marianensis Usinger, 1946: 66; Carvalho 1956: 96 (syn. by Carvalho 1987: 174).
Tinginotum knowlesi Carvalho, 1987: 174; Eyles 2000: 115; Chérot et al. 2017: 112; Chérot 2018: 90; Kim et al. 2025: 562; Chérot et al. 2025: 245.

##### Material examined.

• 1♀, Vietnam, Hue City, Phu Loc, Bach Ma National Park (16.2281°N, 107.8583°E), 1250 m altitude, 6.ix.2025, by light trap, *Kim J*. leg. **[ZCDTU]**; • 1♂, 4♀♀, 1?, Cambodia, Siam Reap Province, Angkor, Preah Khan Temple, 20-27.i.2005, *Var I*. leg.(FC n°s 11547, 11549, 11551, 11553-11555) [**ISNB**]; • 1♀, Cambodia, Siam Reap Province, Angkor, Preah Khan Temple, 21-27.ii.2006, *Var I*. leg. (FC n° 11550) [**ISNB**]; • 1♂, Cambodia, Siam Reap Province, Angkor, Thom, 26.v.2003, *Smets & Grootaert* leg. (FC n° 11548) [**ISNB**]; • 1♀, Cambodia, Siam Reap Province, Angkor, Preah Khan Temple, 24.i-21.ii.2006, *Yothin O*. leg. (FC n° 11552) [**ISNB**].

##### Diagnosis.

Recognized by dorsum greyish brown with brown markings; clypeus mostly pale brown, concolorous with mandibular and maxillary plates; antennae shorter than body; first antennal segment relatively short, subequal to 2× vertex width, pale brown with lateral dark stripe; second antennal segment mostly dark brown except for pale base, middle part and apex; third segment mostly dark brown except for pale extreme apex; labium reaching mid coxae; pronotum somewhat elongate, mostly greyish brown with faint dark markings; scutellum mostly brown, with pale longitudinal line and apex; hemelytra greyish with large brown markings; clavus mostly dark brown with pale small markings; embolium broad; femur pale with dark stripes apically; tibiae pale brown with dark bands and with lateral stripes; tarsus mostly dark brown.

##### Description.

**Figure 5.**
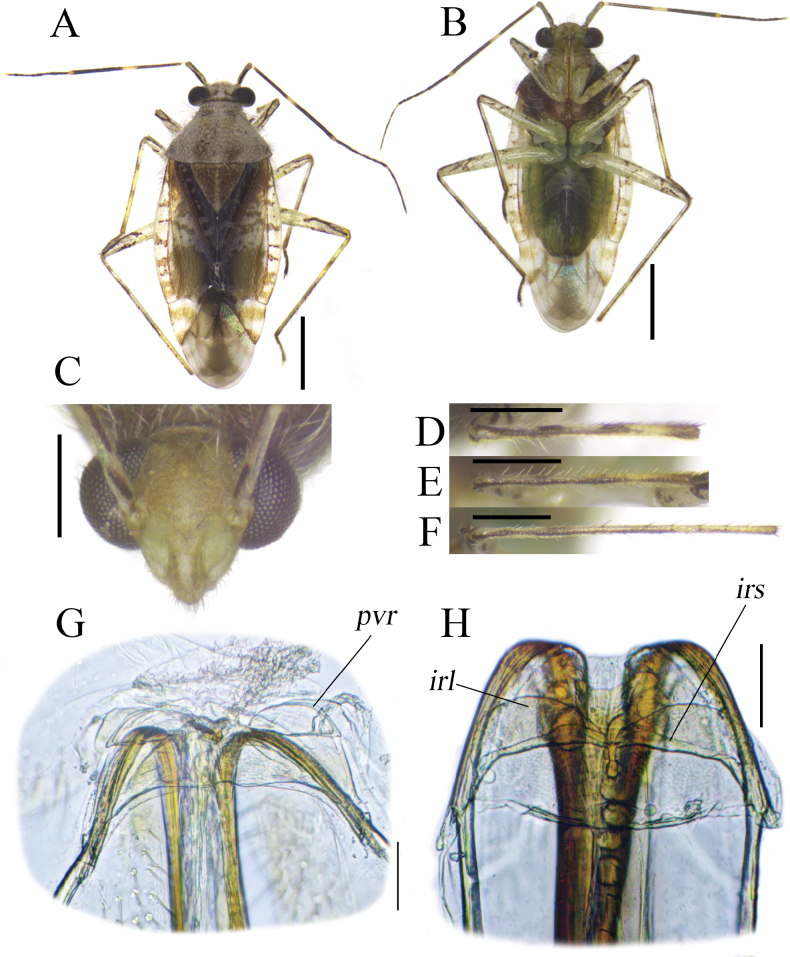
Habitus, diagnostic and female genitalic characters of *Tinginotum knowlesi*. **A, B**. Dorsal and ventral habitus; **C**. Head in frontal view; **D**. Fore tibia; **E**. Mid tibia; **F**. Hind tibia; **G**. Genital chamber; **H**. Posterior wall. Abbreviations: *irl* = interramal lobe; *irs* = interramal sclerite; *pvr* = parieto-vaginal rings. Scale bars: 1 mm (**A, B**); 0.5 mm (**C–F**); 0.1 mm (**G, H**).

See Carvalho (1987) for the most detailed description. As the morphological characters have been well documented except for the female genitalia (Carvalho 1987; Chérot et al. 2025), we describe the female genitalia herein: parieto-vaginal rings (*pvr*) elongate oval, inner margin somewhat blunt, outer margins tapered, anterior and outer margins thick (Fig. 5G); posterior wall with relatively simple interramal sclerite (*irs*) and interramal lobe (*irl*); *irs* generally narrow and broader medially, separated, curved at lateral margin; *irl* broad, entirely covering *irs* and extending further, separated and gradually narrowed medially; sigmoid process elongate (Fig. 5H).

##### Measurements.

Female (*n* = 1) body length, clypeus–apex of membrane: 4.22; head length, excluding collar: 0.35; head width, including compound eyes: 0.92; vertex width: 0.31; first antennal segment length: 0.60; second antennal segment length: 1.57; third antennal segment length: 0.92; fourth antennal segment length: 0.67; total antennal length: 3.76; mesial pronotal length in dorsal view: 0.91; posterior pronotal maximal width (straight): 1.46; anterior scutellar width: 0.79; mesial scutellar length: 0.68; claval commissure length: 0.76; maximal width across hemelytron: 0.87.

##### Plant association.

*Artocarpus altilis*, *A. incisa* (Moraceae), Tobacco-leaves (Solanaceae) (Carvalho and Wallerstein 1976; Eyles 2000).

##### Distribution.

Cambodia*, Caroline Islands, Fiji, Guam, Mariana Islands, New Guinea, New Zealand, Society Islands, Vietnam.

#### Tinginotum perlatum

Taxon classificationAnimaliaHemipteraMiridae

Linnavuori, 1961

5E059B92-B125-52AD-8089-DE317F900191

Figs 6, 7

Tinginotum perlatum Linnavuori, 1961: 155; Yasunaga 1999: 44; Zheng and Lu 2002: 505; Zheng et al. 2004: 587; Oh et al. 2018: 482; Yasunaga 2023: 58; Yasunaga et al. 2023: 38.

##### Material examined.

• 13♂♂17♀♀, Korea, Jeju-do (Is.), Seogwipo-si, Jungmun-dong, 20.ix.2014, *Kim J*. leg., by light trap [CNU]; • 1♂, Korea, Jeju-do (Is.), Seogwipo-si, Donnaeko, 3.ix.2015, *Kim J*. leg. [CNU]; • 1♂2♀♀, Korea, Jeollanam-do, Wando-gun, Gunoe-myeon, Daemun-ri, 18.viii.2015, *Kim J*. leg., by light trap [CNU]; • 1♂, Indonesia, Sumatra, North Sumatra, Berastagi, near Sibayak, 20-26.iv.1998, 1500 m., *Kabourk Vit*. Leg. (FC n° 7447); • 2♀♀, Indonesia, Sumatra, North Sumatra, Berastagi, 02-04.iv.1998, 1500-2000 m., *Kabourk Vit*. Leg. (FC n°s 7444-7445) [Coll. Magnien]; • 2♀♀, Indonesia, Sumatra, North Sumatra, Kedah env., 19.iv.1998, 1500–2000 m., *Kabourk Vit*. Leg. (FC n°s 7446, 7748); • 3♂♂, Philippines, Mindanao Is., Mt. Kitanglad Range, Mt. Kitanglad, 12-14.xi.2018, *Roca-Cusachs M. & Mohagan A*. leg. [ZCDTU]; 1♂, • 5♀♀, Philippines, Luzon, Benguet Province, Baguio, 16.4167°N, 120.6000°E, 07-19.iv.2014, *Constant J., Bresseel, J. & Co* leg. (FC n°s 11541-11546) [ISNB]; • 1♀, Vietnam, Vinh Puc Province, Tam Dao, 17-21.v.1990, *Pacholatko P*. leg. (FC n° 1191) [NHMW].

##### Diagnosis.

**Figure 6.**
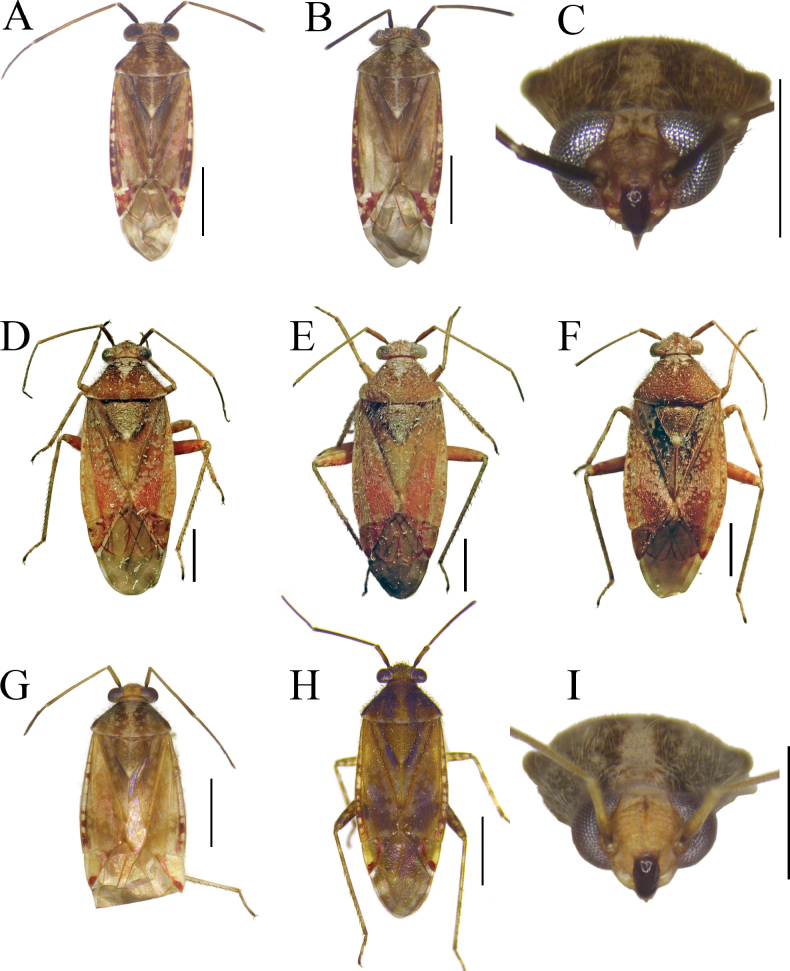
Habitus and head of *Tinginotum perlatum*. **A, B**. Male, in dorsal view; **D–H**. Female, in dorsal view; **C, I**. Head in frontal view; **A–E**. Specimens collected from Philippines; **F**. Specimen collected from Vietnam; **G–I**. Specimens collected from Korea. Scale bars: 1 mm.

Recognized by dorsum brown with reddish areas (Fig. 6A, B, D–H), sometimes tending towards relatively homogenous yellowish, pinkish, and reddish-brown in females; vertex and frons yellowish-brown to brown; clypeus dark brown to almost black, shining, at least on ventral third, contrasting with mandibular and maxillary plates; antennae shorter than body; first antennal segment relatively short, yellowish-brown to dark brown, second antennal segment yellowish brown to dark brown, apex darker, extreme apex pale yellow; third and fourth antennal segments mostly dark brown; pronotum somewhat elongate, with mostly chocolate brown to reddish-brown fuzzy markings behind eyes, a medial longitudinal area generally paler, at least basally; anterior area to humeral angles frequently darker; posterior margin narrowly yellowish; scutellum mostly brown, frequently with pale longitudinal line and apex; hemelytra chocolate brown to orange-brown with reddish or pinkish markings and partly coalescent greyish or silvery spots, faint, faded or even absent in some females; embolium and a part of exocorium sometimes yellowish; cuneus tinged with red; membrane greyish-brown, veins reddish; legs pale brown, metafemora reddish brown apically; metatibial spines yellowish-brown; tarsi yellowish-brown, apex of third segment black.

##### Description.

**Figure 7.**
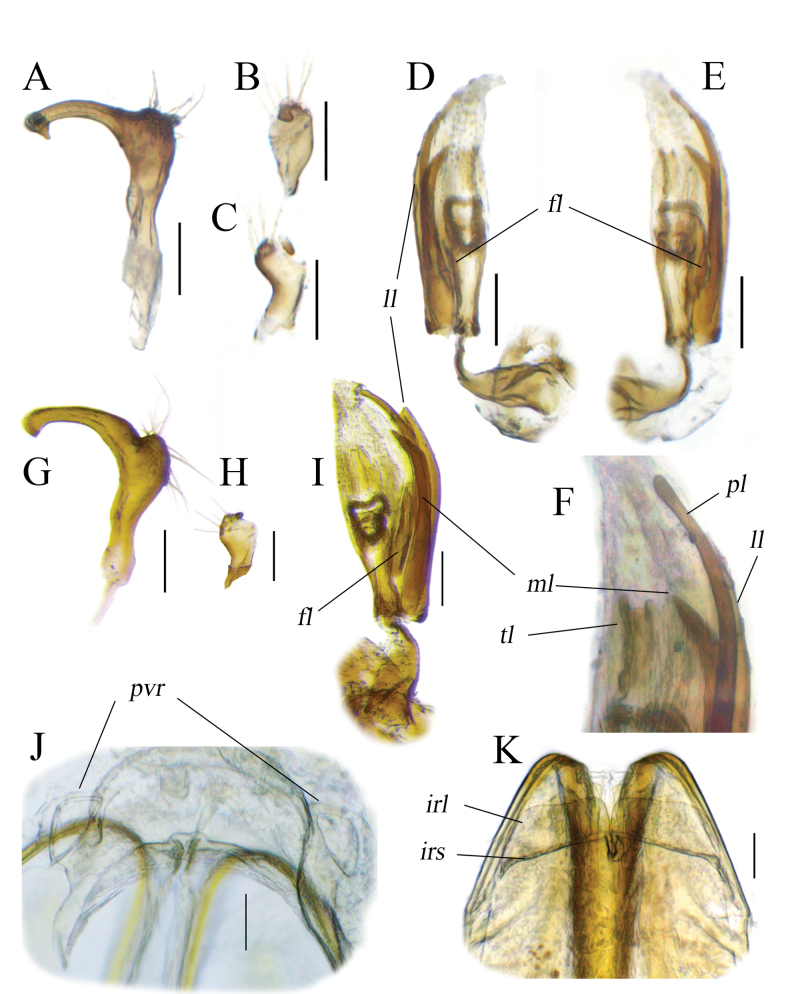
Male (**A–F**) and female (**J–K**) genitalia of *Tinginotum perlatum*. **A, G**. Left paramere; **B, C, H**. Right paramere; **D–F, I**. Endosoma; **J**. Genital chamber; **K**. Posterior wall; **A–F**. Male from Philippines; **G–K**. Male and female genitalia from Korea. Abbreviations: *fl* = fourth sclerite; *irl* = interramal lobe; *irs* = interramal sclerite; *ll* = lateral lobe-like sclerite; *ml* = median sclerite; *pl* = primary sclerite; *pvr* = parieto-vaginal rings; *tl* = third sclerite. Scale bars: 0.1 mm.

See Linnavuori (1961) for most detailed description. As the morphological characters have been well documented except for the female genitalia, we describe the female genitalia here: parieto-vaginal rings (*pvr*) elongate oval, latero-outer and latero-inner margins difficult to separate from anterior and posterior margins, pointed; anterior margins thick, convex, angled, posterior margin convex (Fig. 7J); posterior wall with relatively simple interramal sclerite (*irs*) and interramal lobe (*irl*); *irs* nearly equally narrow, separated, curved at lateral margin; *irl* broad, entirely covering *irs* and extending further, separated from outer margin weakly sinuate; sigmoid process short (Fig. 7K).

##### Measurements.

Male (*n* = 1)/female (*n* = 2) from Luzon, Philippines. Body length, clypeus–apex of membrane: 4.10/6.70–6.80; head length, excluding collar: 0.25/0.50–0.50; head width, including compound eyes: 0.95/1.30–1.33; vertex width: 0.25/0.50–0.50; first antennal segment length: 0.45/0.60–0.60; second antennal segment length: 1.55/2.10–2.20; third antennal segment length: 0.70/1.15–1.25; fourth antennal segment length: 0.60/0.85–0.90; total antennal length: 3.30/4.70–4.95; mesial pronotal length in dorsal view: 0.75/1.00–1.15; posterior pronotal maximal width (straight): 1.40/2.05–2.20; anterior scutellar width: 0.70/1.10–1.10; mesial scutellar length: 0.60/1.00–1.05; maximal width across hemelytron: 0.8/1.12–1.19.

Male (*n* = 3) from Mindanao, Philippines. Body length, clypeus–apex of membrane: 4.14–4.52; head length, excluding collar: 0.30–0.32; head width, including compound eyes: 0.99–1.02; vertex width: 0.24–0.26; first antennal segment length: 0.51–0.59; second antennal segment length: 1.64–1.77; third antennal segment length: 0.87/missing; fourth antennal segment length: missing; mesial pronotal length in dorsal view: 0.74–0.95; posterior pronotal maximal width (straight): 1.40–1.58; anterior scutellar width: 0.69–0.79; mesial scutellar length: 0.70–0.71; claval commissure length: 0.79–0.84; maximal width across hemelytron: 0.76–0.83.

Male (*n* = 1)/Female (*n* = 3) from Korea. Body length, clypeus–apex of membrane: 4.77/5.01–5.10; head length, excluding collar: 0.28/0.38–0.39; head width, including compound eyes: 0.94/1.02–1.04; vertex width: 0.38/0.35–0.37; first antennal segment length: 0.61/0.61–0.68; second antennal segment length: 1.81/1.51–1.70; third antennal segment length: 0.71/0.77–0.81; fourth antennal segment length: 0.59/0.60–0.64; total antennal length: 3.72/3.56–3.83; mesial pronotal length in dorsal view: 0.77/0.87–0.89; posterior pronotal maximal width (straight): 1.49/1.64–1.69; anterior scutellar width: 0.74/0.90–0.91; mesial scutellar length: 0.77/0.81–0.83; claval commissure length: 0.94/0.90–0.96; maximal width across hemelytron: 0.89/0.96–1.04.

##### Remarks.

**Figure 8.**
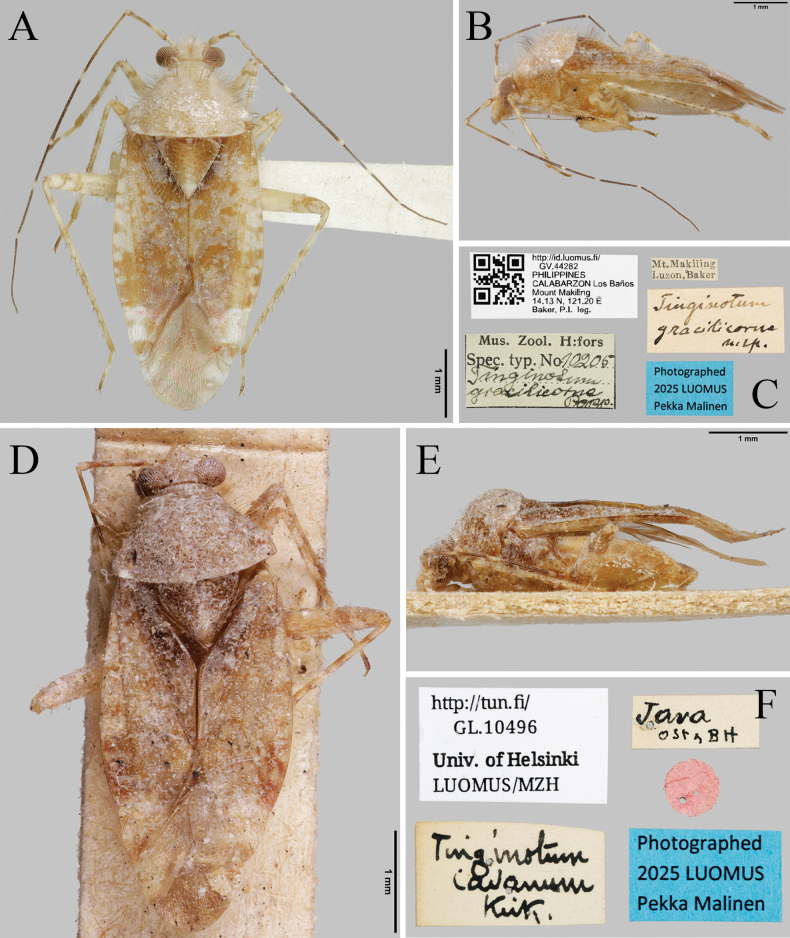
Type specimens and label information of *T. gracilicorne* (**A–C**) and *T. javanum* (**D–F**) (FMNH).

Until now, only *Tinginotum gracilicorne* Poppius, 1915 (Fig. 8A–C) has been recorded from the Philippines (Schuh 2002–2013). The Philippine specimens of *Tinginotum* we have examined can be easily distinguished from *T. gracilicorne*, particularly by the antennal length. Morphologically, we were unable to find any significant differences between these Philippine specimens and *T. perlatum* from Korea and the type locality, Japan.

The only noticeable difference in males is found in the coloration of the cuneus. Based on direct examination of more than 100 individuals from Korea and analysis of pictures of some Japanese specimens (in Yasunaga 2001: fig. 311A; Yasunaga 2023: fig. 213 and Yasunaga et al. 2023: fig. 24G), the cuneus of East-Palaearctic populations is generally subhyaline, with only the inner margin tinged with red, as indicated in the original description of *T. perlatum* (Linnavuori, 1961). In contrast, in the Philippine specimens, the cuneus is largely tinged with red. Interestingly, dorsal coloration within series of specimens from Luzon and Sumatra seems relatively variable, particularly in females (Fig. 6D, E), their pattern being faded, whereas it is very close to that of the males on a female from Vietnam (Fig. 6F). No differences were observed in the male genitalia, except for slight differences in the degree of development of the apex of shaft and the sensory lobe of the left paramere and shaft of the right paramere (Fig. 7A–F).

Although the morphology of the endosoma is very similar in many *Tinginotum* species, based on the available evidence, including general morphology as well as male and female genital structures, we tentatively identify specimens from Indonesia and the Philippines as *T. perlatum*, a species recorded from both countries and from Vietnam for the first time. Further studies are required to confirm whether these populations are truly conspecific and whether *T. perlatum* is effectively a very widely distributed species. In addition, although this species was originally recorded from the Palaearctic Region, its apparently broader distribution raises the possibility that it may be conspecific with taxa previously described from tropical regions. Accordingly, future studies based on broader geographic sampling, including material from the type locality, will be necessary to clarify its taxonomic status.

##### Plant association.

Two coniferous and one fern have been cited in the literature as possible associated plants: *Juniperus chinensis* (Cupressaceae), *Microlepia strigosa* (Polypodiaceae), *Pinus luchensis* (Pinaceae) (Yasunaga 1999). More recently, *T. perlatum* has been reported to utilize male strobili of *Cryptomeria japonica* (Cupressaceae) as a food resource, and its feeding activity has been associated with increased mortality of male strobili and reduced pollen production (Muro 2025).

##### Distribution.

China, Indonesia (Sumatra)*, Japan, Korea, Taiwan, Philippines (Luzon, Mindanao)*, Vietnam (northern; Vinh Phuc)*.

#### Tinginotum gracilicorne

Taxon classificationAnimaliaHemipteraMiridae

Poppius, 1915

EE8ADC10-3A6B-59E7-9306-0F2DF85F6883

Fig. 8A–C

Tinginotum gracilicorne Poppius, 1915: 57; Carvalho 1959: 267.

##### Material examined.

Further details on the specimen are available on the online portal of the holding institution (FMNH) at http://id.luomus.fi/GV.44282.

##### Distribution.

Philippines (Luzon).

##### Remarks.

The following characters may serve as diagnostic features of this species: clypeus mostly pale brown; antennae mostly dark brown with pale parts, long and slender; first segment partially pale and brown, basal half strongly swollen and yellowish, apical half brown; second segment mostly dark brown, 1/3 basal part, middle and apex pale, slightly more than twice as long as first antennal segment; labium reaching middle coxae; scutellum mostly brown, somewhat weakly punctate, more sparsely punctate than pronotum; femora pale with brown bands, one band medially and two bands apically; tibiae pale with brownish bands.

This species resembles *T. formosanum* and *T. knowlesi* in general body form and overall antennal color pattern but differs in having elongate antennae. It can be distinguished from *T. formosanum* by the color pattern of the second antennal segment, by the absence of dark tufts of setae on the pronotum, and by the relatively weaker punctation on the scutellum. From *T. knowlesi*, it differs in lacking stripes on the first antennal segment, in the coloration pattern of the second antennal segment, and in the absence of apical stripes on the femora and tibiae.

#### Tinginotum javanum

Taxon classificationAnimaliaHemipteraMiridae

Kirkaldy, 1902

A04674EB-1E55-5621-AABC-27B4C1B33288

Fig. 8D–F

Tinginotum javanum Kirkaldy, 1902: 263; Poppius 1911: 22; Carvalho 1952: 93; 1959: 268.

##### Material examined.

• 1♂, Indonesia, Borneo, South Kalimatan, Loksados (7 km NE), 22.ix.1997, *Jakl S*. leg. (FC n° 11556) [**ISNB**]. Further details on the specimen are available on the online portal of the holding institution (FMNH) at https://kotka.luomus.fi/view?uri=luomus:GL.10496.

##### Distribution.

Indonesia (Borneo, Java), Sri Lanka (Carvalho 1959).

##### Remarks.

This species is the type species of the genus *Tinginotum*. In the original description, it was described together with the generic description of *Tinginotum*, and therefore its species-level description is relatively simple. Although only a few morphological characters can be examined from images of the type specimen, the coloration pattern of the antennae, the relative proportions of the antennal segments (second segment twice as long as the third, which is slightly longer than the first, with the three apical segments slender), and the mostly pale brown clypeus may be considered diagnostic for this species.

This species resembles *T. formosanum*; however, *T. formosanum* possesses distinct clusters of dark setae on the pronotum, whereas such a feature is neither mentioned in the original description of this species nor observable in the type specimen. According to Poppius (1915), it can be distinguished by the proportions of the antennal segments and by the relatively weak punctation on the scutellum, but because the type specimen of *T. javanum* is in poor condition, reliable comparison is difficult, and additional material will be needed to confirm these characters.

#### Tinginotum kirkaldyi

Taxon classificationAnimaliaHemipteraMiridae

Poppius, 1914

7C987FB4-37AE-555B-BEAC-94D7498BBA3E

Fig. 9A–D

Tinginotum kirkaldyi Poppius, 1914: 117; Carvalho 1959: 268.

##### Material examined.

Further details on the specimen are available on the online portal of the holding institution (FMNH) at http://id.luomus.fi/GV.44279.

##### Distribution.

Indonesia (Java).

##### Remarks.

The following characters may serve as diagnostic features of this species: clypeus entirely dark brown; antennae mostly dark brown, clearly shorter than body; first segment mostly pale brown with dark ring basally; second segment mostly dark brown, darkened toward apex, slightly > 3 × as long as first antennal segment length; labium reaching middle coxae; scutellum flat, punctate to degree similar to that of pronotum; cuneus largely with reddish markings; tibiae pale with brownish bands.

**Figure 9.**
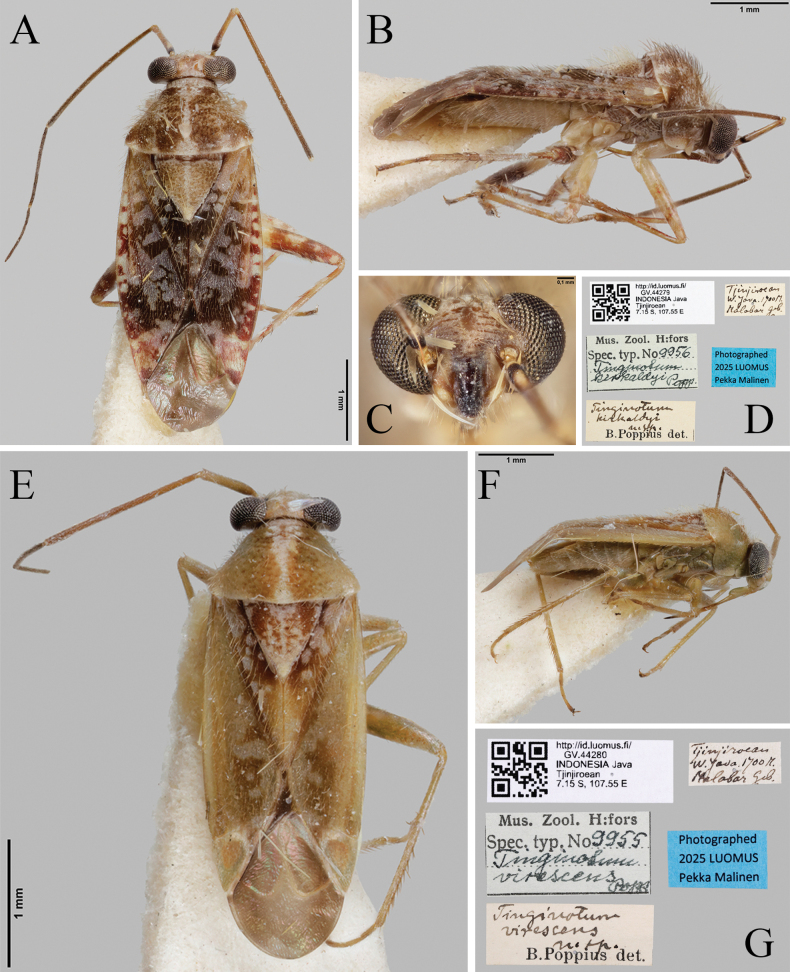
Type specimens and label information of *T. kirkaldyi* (**A–D**) and *T. virescens* (**E–G**) (FMNH).

This species is similar to *T. perlatum* but can be distinguished by the presence of a distinct dark band on the hind tibia and by the broadly developed red marking on the cuneus. It also resembles the Australian species *T. minutum*, but differs in having the first antennal segment generally pale brown with a dark basal ring and the second antennal segment mostly dark brown, becoming darker toward the apex.

#### Tinginotum virescens

Taxon classificationAnimaliaHemipteraMiridae

Poppius, 1914

D1D4C897-396E-5B57-82B1-FA81D207C46A

Fig. 9E–G

Tinginotum virescens Poppius, 1914: 118; Carvalho 1959: 268.
Tinginotum kandanensis Carvalho, 1987: 172 (syn. by Yasunaga 1999).

##### Material examined.

Further details on the specimen are available on the online portal of the holding institution (FMNH) at http://id.luomus.fi/GV.44280.

##### Distribution.

Indonesia (Java), Papua New Guinea.

##### Remarks.

This species resembles *T. kirkaldyi* and *T. perlatum* in general body form and pronotal pattern but can be distinguished by its fading coloration (greenish brown body in original description), smaller body size, generally pale-brown clypeus, the absence of markings on the embolium, and the brown coloration on the central part of the cuneus.

### Type photographs of some Southeast Asian *Tinginotum* species

**Figure 10.**
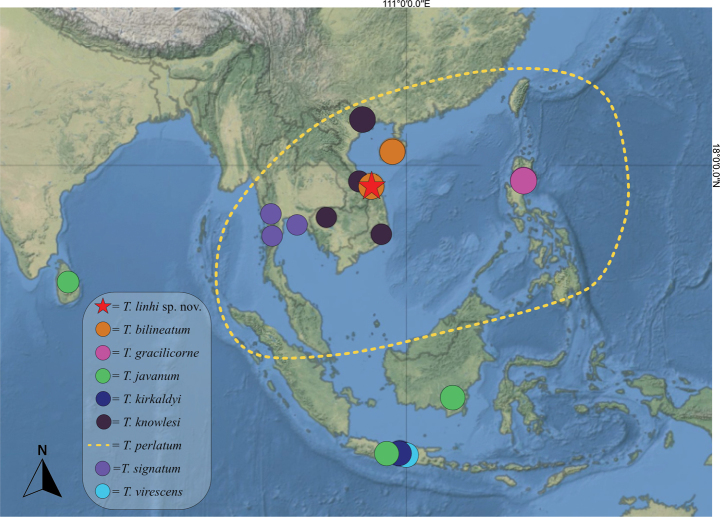
Distributional map of *Tinginotum* species addressed in this study. Yellow dashed line indicates the potential distribution of *T. perlatum* based on currently available records.

In the present study, we aimed to include photographs of the type specimens that we have obtained, as limited information is currently available on the species recorded from Southeast Asia, except for the original descriptions. However, since the original descriptions are relatively well written and we did not directly examine these specimens, we do not duplicate the descriptions in this paper. Instead, we include only photographs of the type specimens for each species, accompanied by a brief summary of the characters extracted from both the original descriptions and image of the type specimens, as well as distributional data (Fig. 10), with the hope that these data may be helpful for future research.

## Supplementary Material

XML Treatment for Tinginotum

XML Treatment for Tinginotum linhi

XML Treatment for Tinginotum bilineatum

XML Treatment for Tinginotum knowlesi

XML Treatment for Tinginotum perlatum

XML Treatment for Tinginotum gracilicorne

XML Treatment for Tinginotum javanum

XML Treatment for Tinginotum kirkaldyi

XML Treatment for Tinginotum virescens

## Additional information

### Use of AI

No use of AI was reported.

### Author ORCIDs

Junggon Kim https://orcid.org/0000-0003-0594-7618

Frédéric Chérot https://orcid.org/0000-0001-7918-7012

Quoc Toan Phan https://orcid.org/0000-0002-3154-6546

Minh Ty Nguyen https://orcid.org/0000-0002-2023-1813

Ba Vu Lam Nguyen https://orcid.org/0009-0009-6166-9705

Sunghoon Jung https://orcid.org/0000-0001-6086-0326

### Data availability

All of the data that support the findings of this study are available in the main text.

## References

[B1] Carvalho JCM (1952) On the major classification of the Miridae (Hemiptera). (With keys to subfamilies and tribes and a catalogue of the world genera). Anais da Academia Brasileira de Ciências 24: 31–110.

[B2] Carvalho JCM (1956) Insects of Micronesia: Miridae. Bishop Museum, Honolulu 7: 1–100.

[B3] Carvalho JCM (1959) A catalogue of the Miridae of the world. Part IV. Arquivos do Museu Nacional, Rio de Janeiro 48: 1–384.

[B4] Carvalho JCM (1987) The genera *Tinginotopsis* Poppius and *Tinginotum* Kirkaldy from Papua New Guinea (Hemiptera, MIridae). Revista Brasileira de Biologia 47: 165–176.

[B5] Carvalho JCM, Wallerstein P (1976) On Kirkaldy’s types of *Felisacus filicicola*, *Nesodaphne knowlesi* and *Pseudoloxops vitiensis* (Hemiptera, Miridae). Revista Brasileira de Biologia 36: 687–691.

[B6] Cheesman LE (1926) A new genus and species of Miridae (Hemiptera) from the Society Islands. Entomologist 59: 266–267. 10.1111/j.1365-2311.1927.tb00068.x

[B7] Cheesman LE (1927) A contribution towards the insect fauna of French Oceania. Part I. The Transactions of the Entomological Society of London 75: 147–161.

[B8] Chérot F (2018) Miscellanea Miridologica V. Taxonomy and chorology of new or little known taxa of continental New Guinea and neighboring islands (Insecta, Heteroptera, Miridae). ZooKeys 796: 83–95. 10.3897/zookeys.796.20736 PMC6250776 30487711

[B9] Chérot F, Gorczyca J, Schwartz MD, Demol T (2017) The Bryocorinae, Cylapinae, Deraeocorinae and Mirinae (Insecta: Heteroptera: Miridae) from Baiteta Forest, Papua New Guinea, with a discussion of their feeding habits and a list of species of the country. In: Telnov D, Barclay MVL, Pauwels OSG (Eds) Biodiversity, Biogeography and Nature Conservation in Wallacea and New Guinea. Vol. III. The Entomological Society of Latvia, Riga, 55–139.

[B10] Chérot F, Yasunaga T, Schwartz MD, Carapezza A, Carpintero DL, Yeshwanth HM (2025) The genera of dorsally punctate Mirini and some related extralimital taxa: Short diagnosis and synthesis of available data with a focus on the so-called *Lygus* complex (Hemiptera: Heteroptera: Miridae). Zootaxa 5672(1): 001–559. 10.11646/zootaxa.5672.1.1 41119452

[B11] Distant WL (1904) The fauna of British India, including Ceylon and Burma. Rhynchota. Taylor & Francis, London. Vol. 2, part 2, 243–503.

[B12] Distant WL (1913) Reports of the Percy Sladen Trust Expedition to the Indian Ocean in 1905. No. IX Rhynchota. Part I: Suborder Heteroptera. Transactions of the Linnaean Socity of London 16: 139–190, plates 11–13. 10.1111/j.1096-3642.1913.tb00115.x

[B13] Eyles AC (2000) *Tinginotum* Kirkaldy in New Zealand and Australia: a shared new species, and a new species of *Tinginotopsis* Poppius from Norfolk Island (Hemiptera: Miridae). New Zealand Journal of Zoology 27: 111–119. 10.1080/03014223.2000.9518216

[B14] Kerzhner IM (1972) New and little known Heteroptera from the Far East of the USSR. Trudy Zoolologicheskogo Instituta Akademiya Nauk SSSR 52: 276–295.

[B15] Kim J, Jung S (2019) Phylogeny of the plant bug subfamily Mirinae (Hemiptera: Heteroptera: Cimicomorpha: Miridae) based on total evidence analysis. Systematic Entomology 44(4): 686–698. 10.1111/syen.12348

[B16] Kim J, Chérot F, Phan QT, Jung S (2025a) Two new species of the tribe Mirini (Hemiptera: Heteroptera: Miridae: Mirinae) from Vietnam, with additional data to Vietnamese Mirinae catalogue. Zootaxa 5606(4): 555–564. 10.11646/zootaxa.5604.4.7 40174182

[B17] Kim J, Chérot F, Phan QT, Keetapithchayakul TS, Jung S (2025b) A Catalogue of the Subfamily Mirinae (Insecta: Hemiptera: Heteroptera: Miridae) of Vietnam. Journal of the International Heteropterist’s Society 2(1): 23–37. 10.11646/jihs.2.1.3

[B18] Kirkaldy GW (1902) Memoir upon the Rhyncotal family Capsidae Auctt. The Transactions of the Entomological Society of London 1902: 243–272. [pls V, VI.] 10.1111/j.1365-2311.1902.tb01384.x

[B19] Kirkaldy GW (1908) A Catalogue of the Hemiptera of Fiji. Proceedings of the Linnean Society of New South Wales 33: 345–391.

[B20] Kulik SA (1965) New species of capsid-bugs (Heteroptera, Miridae) from East Siberia and from the Far East. Zoologicheskii jurnal [Journal of Zoology] 44: 1497–1505. [in Russian]

[B21] Linnavuori RE (1961) Contributions to the Miridae fauna of the Far East. Annales Entomologici Fennici 27: 155–169.

[B22] Muro T (2025) The relationship between sucking behavior and population density of *Tinginotum perlatum* (Hemiptera: Miridae), and mortality rate of male strobili of *Cryptomeria japonica* in miniature seed orchard. Tree and Forest Health 29: 139–143.

[B23] Namyatova AA, Schwartz MD, Cassis G (2021) Determining the position of *Diomocoris*, *Micromimetus* and *Taylorilygus* in the *Lygus* complex based on molecular data and first records of *Diomocoris* and *Micromimetus* from Australia, including four new species (Insecta: Hemiptera: Miridae: Mirinae). Invertebrate Systematics 35: 90–131. 10.1071/IS20015

[B24] Odhiambo TR (1960) Notes on East African Miridae (Hemiptera). XIX. *Tinginotum* Kirkaldy. Annals & Magazine of Natural History 3(13): 465–489. 10.1080/00222936008651045

[B25] Oh M, Yasunaga T, Duwal RK, Lee S (2018) Annotated checklist of the plant bug tribe Mirini (Heteroptera: Miridae: Mirinae) recorded on the Korean Peninsula, with descriptions of three new species. European Journal of Entomology 115: 467–492. 10.14411/eje.2018.048

[B26] Poppius B (1911) Beiträge zur Kenntnis der Miriden-Fauna von Ceylon. Öfversigt af Finska Vetenskapssocietetens Förhandlingar 53A(2): 1–36.

[B27] Poppius B (1912) Die Miriden der Äthiopischen Region I Mirina, Cylapina, Bryocorina. Acta Societatis Scientiarum Fennicae 41(3): 1–203 [one plate]. 10.5962/bhl.title.53693

[B28] Poppius B (1914) Zur Kenntnis der Miriden, Anthocoriden und Nabiden Javas und Sumatras. Tijdschrift voor Entomologie 56 [suppl.]: 100–187.

[B29] Poppius B (1915) Zur Kenntnis der indo-australischen Capsarien. I. Annales Historico-Naturales Musei Nationalis Hungarici 13: 1–89.

[B30] Schuh RT (2002–2013) Online Systematic Catalog of Plant Bugs (Insecta: Heteroptera: Miridae). The American Museum of Natural History. http://research.amnh.org/pbi/catalog/ [accessed 1 Nov 2025]

[B31] Usinger RL (1946) HemipteraHeteroptera of Guam. In: Insects of Guam. II. Bulletin of the Bishop Museum 189: 11–103.

[B32] Yasunaga T (1999) Revision of the mirine genus *Tinginotum* Kirkaldy (Heteroptera: Miridae) from Japan. Biogeography 1: 39–47.

[B33] Yasunaga T (2001) Family Miridae Hahn, plant bugs. In: Yasunaga T, Takai M, Kawasawa T, Nakatani Y (Eds) A field Guide to Japanse Bugs. II. Terrestrial Heteropterans. Anthocoridae, Cimicidae, Microphysidae and Miridae. Zenkoku Noson Kyoiku Kyokai, Publishing Co., Ltd., Tokyo, 2–96, 111–351. [in Japanese]

[B34] Yasunaga T (2023) Descriptions of twenty-three new mirine species from Japan, with a key to genera of the tribe Mirini, updating the Japanese fauna (Hemiptera: Miridae: Mirinae). Tijdschrift voor Entomologie 166: 1–116. 10.1163/22119434-bja10024

[B35] Yasunaga T, Schwartz MD, Chérot F (2023) Revision of the plant bug genus *Diognetus*, with descriptions of thirteen new species from the Oriental and Eastern Palearctic Regions (Hemiptera: Heteroptera: Miridae). Acta Entomologica 63(1): 1–55. 10.37520/aemnp.2023.001

[B36] Zheng LY, Lu N (2002) On *Orthops* Fieber and some new species of Mirinae from China (Hemiptera: Miridae). Acta Zootaxonomica Sinica 27: 498–507.

[B37] Zheng LY, Lu N, Liu G, Xu B (2004) Hemiptera. Miridae. Mirinae. Fauna Sinica. Insecta Vol. 33. Chinese Academy of Sciences. Science Press, Beijing, China, 797 pp.

